# The “elongate chelicera problem”: A virtual approach in an extinct pterygotid sea scorpion from a 3D kinematic point of view

**DOI:** 10.1002/ece3.11303

**Published:** 2024-05-17

**Authors:** Michel Schmidt, Roland R. Melzer

**Affiliations:** ^1^ Yunnan Key Laboratory for Palaeobiology Yunnan University Kunming China; ^2^ MEC International Joint Laboratory for Palaeobiology and Palaeoenvironment Yunnan University Kunming China; ^3^ Bavarian State Collection of Zoology Bavarian Natural History Collections München Germany; ^4^ Ludwig‐Maximilians‐University Munich Faculty of Biology Biocentre Munich Germany; ^5^ GeoBio‐Center Ludwig‐Maximilians‐Universität München München Germany

**Keywords:** 3D kinematics, *Acutiramus*, cheliceres, Eurypterida, Pterygotidae, sea scorpions, virtual paleontology

## Abstract

Chelicerae, distinctive feeding appendages in chelicerates, such as spiders, scorpions, or horseshoe crabs, can be classified based on their orientation relative to the body axis simplified as either orthognathous (parallel) or labidognathous (inclined), exhibiting considerable diversity across various taxa. Among extinct chelicerates, sea scorpions belonging to the Pterygotidae represent the only chelicerates possessing markedly elongated chelicerae relative to body length. Despite various hypotheses regarding the potential ecological functions and feeding movements of these structures, no comprehensive 3D kinematic investigation has been conducted yet to test these ideas. In this study, we generated a comprehensive 3D model of the pterygotid Acutiramus, making the elongated right chelicera movable by equipping it with virtual joint axes for conducting Range of Motion analyses. Due to the absence in the fossil record of a clear indication of the chelicerae orientation and their potential lateral or ventral movements (vertical or horizontal insertion of joint axis 1), we explored the Range of Motion analyses under four distinct kinematic settings with two orientation modes (euthygnathous, klinogathous) analogous to the terminology of the terrestrial relatives. The most plausible kinematic setting involved euthygnathous chelicerae being folded ventrally over a horizontal joint axis. This configuration positioned the chelicera closest to the oral opening. Concerning the maximum excursion angle, our analysis revealed that the chela could open up to 70°, while it could be retracted against the basal element to a maximum of 145°. The maximum excursion in the proximal joint varied between 55° and 120° based on the insertion and orientation. Our findings underscore the utility of applying 3D kinematics to fossilized arthropods for addressing inquiries on functional ecology such as prey capture and handling, enabling insights into their possible behavioral patterns. Pterygotidae likely captured and processed their prey using the chelicerae, subsequently transporting it to the oral opening with the assistance of other prosomal appendages.

## Graphical abstract

How pterygotid sea scorpions used their tripartite elongate cheliceres to get prey items close to the mouth region remains a conundrum. We applied four different 3D kinematic settings with regard to insertion and orientation of the cheliceres to examine the most likely way this was realized. We found orthognathous cheliceres with a horizontal proximal joint axis allowing for a ventral movement the most logical to have been established.

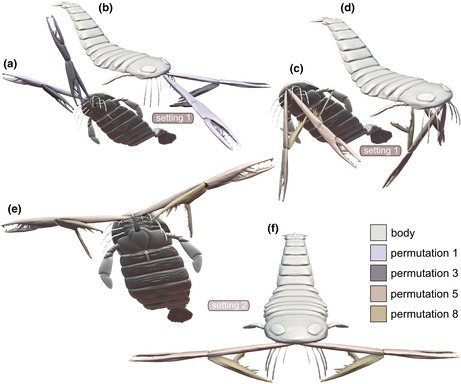

## 1 INTRODUCTION

Chelicerates represent a diverse assemblage of euarthropods encompassing both Pycnogonida, denoting aquatic sea spiders, and Euchelicerata. The latter includes extant Arachnida, featuring terrestrial taxa, such as spiders (Araneae), harvestmen (Opiliones), scorpions (Scorpiones), sun spiders (Solifugae), aquatic mites (among Acariformes, Parasitiformes), and others. In addition to Arachnida, Euchelicerata incorporates horseshoe crabs (Xiphosura) and the extinct sea scorpions (Eurypterida), along with the morphologically similar Chasmataspidida (Lamsdell et al., 2019). While the systematic affiliations within certain Euchelicerata groups pose challenges, exhibiting considerable disparities in morphological and genetic arrangements in phylogenetic analyses (Ballesteros et al., 2022; Sharma et al., 2021), the overarching characteristic defining the Chelicerata clade is the presence of chelicerae.

Chelicerae represent specialized mouthparts that play pivotal roles in feeding and defense, constituting a crucial factor in comprehending evolutionary adaptations (Foelix et al., 2005). In Pycnogonida, a basal chelicerate group, they are referred to as chelifores, which are thought to be homologous to chelicerae. In diverse taxa, each chelicera is bipartite, comprising a basal element and a terminal element. The basal element affixes to the anterior part of the body (typically the prosoma), providing structural support, while the terminal element encompasses a movable part, such as a fang or claw, serving varied functions like grasping or piercing. Regarding their orientation along the longitudinal body axis, bipartite chelicerae superficially can be distinguished into two primary forms: orthognathous and labidognathous. Orthognathous chelicerae are found in mygalomorph spiders (e.g., tarantulas), mesothele spiders (featuring a segmented opisthosoma), Amblypygi, Thelyphonida, Schizomida, and Solifugae. They are characterized by a straight, parallel alignment with the longitudinal body axis. Labidognathous chelicerae are oriented at a taxon‐specific angle to the longitudinal body axis, allowing for a more versatile Range of Motion (Foelix, 2011), and are exclusive to araneomorph spiders. However, those categories represent simplifications; in fact, there are gradations between them. To reflect the somewhat splayed condition present for instance in mesothele spiders, Kraus and Kraus (1993) even specifically introduced the term “plagiognathy.”

Bipartite chelicerae further exhibit chelate morphology (Solifugae, Ricinulei, Pseudoscorpiones, and Acariformes), resembling a scissor, or a subchelate form (Mygalomorphae, Mesothele, Amblypygi, Thelyphonida, and Schizomida), resembling a jackknife.

Some chelicerates deviate from the standard bipartite arrangement, possessing predominantly chelate chelicerae with three distinct elements, rendering them tripartite (Pycnogonida, Opiliones, Scorpiones, Palpigradi, Parasitiformes, Xiphosura, and the extinct Eurypterida). Given the phylogenetic antiquity of these taxa, the unique structural adaptation raises questions about the evolutionary implications of such variations. Bipartite, subchelate chelicerae as seen in spiders (Araneae) may be considered a derived character, prompting the hypothesis that tripartite chelicerae represent the ancestral condition (e.g., Dunlop, 1996; Giribet et al., 2002; Sharma et al., 2021; Shultz, 2007; Wheeler & Hayashi, 1998).

Concerning overall body size, extant chelicerates generally exhibit diminutive dimensions compared to some Eurypterida. Specific terrestrial arachnid groups such as Theraphosidae do exhibit a leg span of around 20 cm with a body length of only around 10 cm (Marshall & Uetz, 1993). Marine pycnogonids (Vinu et al., 2016) can exhibit a 70 cm leg span, but with a body length of only about 7 cm. While the largest extant aquatic euchelicerates, horseshoe crabs (Lamsdell, 2020), attain body lengths of around 30 cm, the historical pinnacle of arthropod dimensions with cheliceres was achieved by the extinct sea scorpions (Eurypterida). These formidable creatures purportedly reached total body lengths exceeding 200 cm (Braddy et al., 2008), marking a crucial epoch in the evolutionary saga of chelicerates as the pioneering euarthropods documented to possess chelicerae (Clarke & Ruedemann, 1912). While most eurypterid groups manifested relatively modest cheliceres, the Pterygotida, including the potentially largest eurypterid, *Jaekelopterus rhenaniae* (Braddy et al., 2008), stood out with elongated chelicerae that posed challenges to their intricate feeding ecology (Braddy, 2023; Chlupáč, 1994; Kjellesvig‐Waering, 1964).

The Pterygotida, formidable predators in the early Paleozoic seas from the late Silurian (Wenlock) to the mid‐Devonian (Givetian), present an intriguing puzzle for paleontologists. As all documented instances of Eurypterida in the fossil record feature tripartite chelicerae, it is probable that Pterygotida also possessed three‐segmented chelicerae. However, their chelicerae were of such length that they likely encountered numerous challenges in transporting prey to the mouth, given that the basal element exceeded the length of the chela, comprising the free and fixed ramus.

Consequently, the elongated chelicerae of Pterygotida were initially thought to consist of four elements (Clarke & Ruedemann, 1912; Kjellesvig‐Waering, 1964), a notion later revised to three (Bicknell, Kenny, & Plotnick, 2023; Laub et al., 2010). Laub et al. (2010) elucidated potential morpho‐functional implications for the feeding ecology of the pterygotid *Acutiramus* Ruedemann, 1935 having either three‐ or four‐part chelicerae. Hence, they concluded that the presumed “elbow‐joint” described by Kjellesvig‐Waering (1964) likely represented a topographic irregularity.

Upon reanalysis of the same material (BMS E837), Bicknell, Kenny, and Plotnick (2023) demonstrated that the structure was more likely a crack than a joint, challenging the interpretation of Kjellesvig‐Waering (1964) of four elements. Consequently, the current understanding of Pterygotida, specifically *Acutiramus*, posits that their chelicerae comprised only three elements. This limitation might have significantly constrained their Range of Motion. The absence of a distinct “elbow‐joint” complicates our comprehension of their feeding strategies, necessitating advanced techniques such as 3D kinematics in the realm of virtual paleontology.

Numerous questions persist, such as whether the chelicerae exhibited an orientation parallel or straight to the body axis (comparable to an orthognathous state in terrestrial chelicerate relatives) or angled relative to the longitudinal axis of the body (comparable to a labidognathous state in terrestrial chelicerate relatives). Additionally, the direction in which the entire chelicera could move (mediolaterally or dorsoventrally) remains unknown and hinges on whether the presumed bicondylar joint connecting each chelicera to the body possessed a rather vertically or horizontally aligned joint axis.

To distinguish the orientation mode of the chelicerae of extinct marine Pterygotidae from terrestrial chelicerate relatives, we here introduce the following terminology: by euthygnathous (εὐθύς = straight, parallel; γνάθος = jaw), we understand chelicerae with an orientation parallel to the longitudinal axis of the body, by klinognathous (κλινός = inclined; γνάθος = jaw), we understand an orientation at a specific angle relative to the longitudinal axis of the body. This angle we set to 45°.

To elucidate the most plausible chelicerae orientation angle and movement direction that could explain their feeding ecology—particularly in bringing prey items closest to the ventral surface and gnathobasic coxae of the prosomal appendages for further food processing—we employed and modeled based on our introduced terminology four distinct kinematic settings:

1. euthygnathous chelicerae with a horizontal joint axis, allowing for dorsoventral motion;
2. euthygnathous chelicerae with a vertical joint axis, allowing for mediolateral motion;
3. klinognathous chelicerae allowing for an inclined dorsoventral motion;
4. klinognathous chelicerae allowing for an inclined mediolateral motion.

Through the application of 3D kinematics, our objective is to illuminate the functional morphology of *Acutiramus*' chelicerae, providing valuable insights into the likely feeding strategies and ecological adaptations of these mysterious sea scorpions.

## 2 MATERIALS AND METHODS

### 2.1 Three‐dimensional reconstruction of the *Acutiramus* model

The three‐dimensional model of *Acutiramus* sp. was created using Blender version 4.0.1. The model integrates informative morphological data from the original description of *Pterygotus buffaloensis* (referred to as *Acutiramus cummingsi* (Grote & Pitt, 1877), as depicted in Clarke & Ruedemann, 1912, and subsequently in Størmer, 1955). Additionally, we incorporated recent findings on appendicular insertion and orientation, as well as the reduced appendage II, from Bicknell, Kenny, and Plotnick (2023). Although mistakenly Bicknell, Kenny, and Plotnick (2023) introduced a more robust, thicker, and longer *Eurypterus*‐like “swimming leg” (appendage VI) to their reconstruction of the pterygotid *Acutiramus*. In contrast, the Pterygotidae exhibit slender and smaller appendages (Bicknell, Kenny, & Plotnick, 2023, figure 8). To align with the conventional representation of these structures, we modeled them based on Clarke and Ruedemann (1912).

The overall model was scaled to accommodate a 60 cm long single chelicera (Bicknell, Kenny, & Plotnick, 2023, figure 8C). The model was constructed with the free ramus of the chelicera as the exterior, similar to extant *Ischyropsalis* harvestmen, considering the absence of conclusive information in the fossil record regarding its placement (interior or exterior). For visual representation, we modeled a flexed metasoma and flexed appendages. The spacing between the chelicerae and the angle between them were designed in accordance with Bicknell et al. (2023, figure 8), and so was the inflation of the body parts. Upon completion, the model was exported as an .obj file.

### 2.2 Terminology and kinematic settings

**FIGURE 1:**
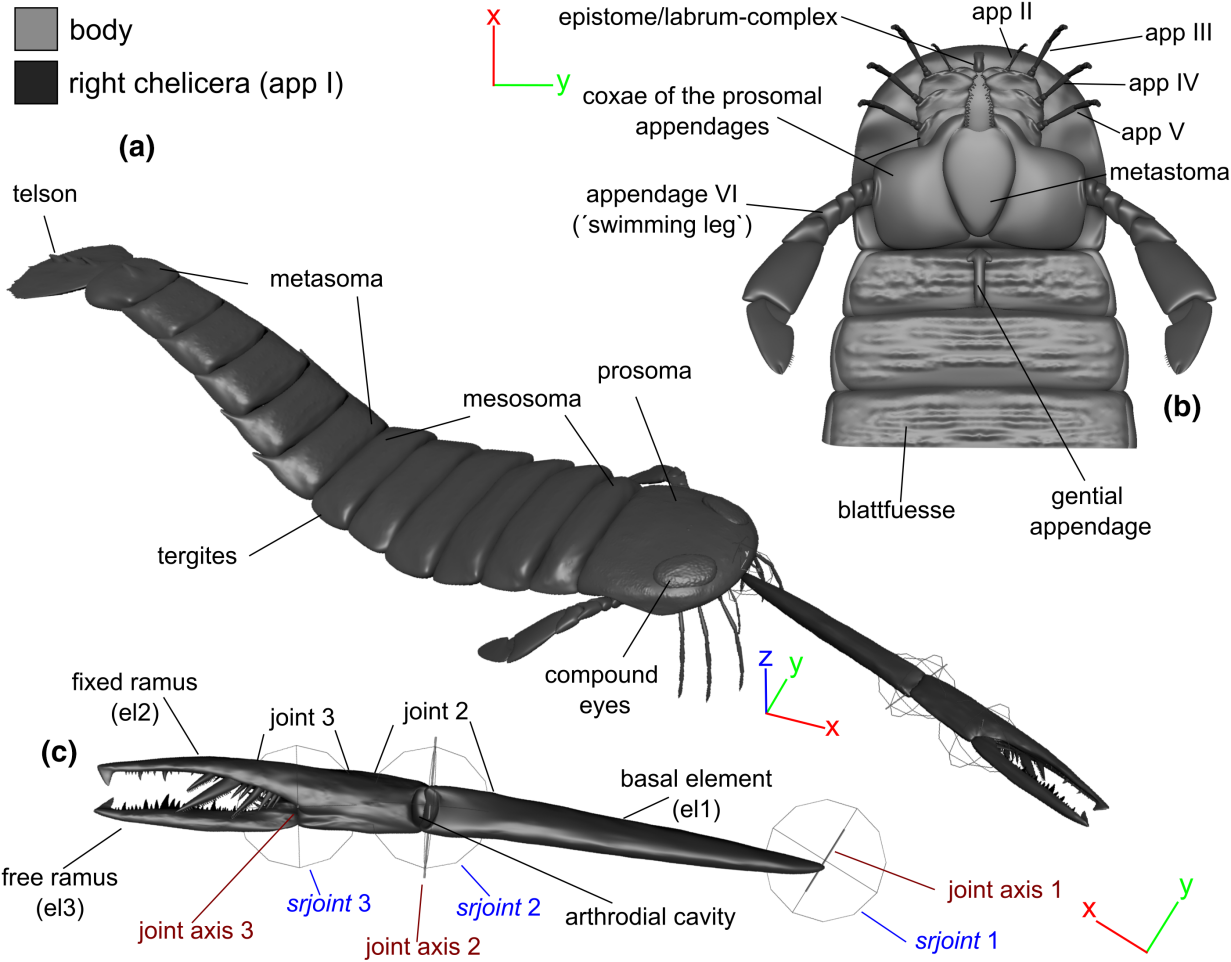
Morphology and terminology. (a) Kinematic representation of the complete *Acutiramus* 3D model (body and right chelicera), anterodorsal oblique view. (b) Elaboration on the ventral morphology of the prosoma (excluding the chelicera), ventral view. (c) Kinematic marionette illustrating the dynamics of the right chelicera ventral view. app *n*, appendage *n*; el1–3, cheliceral element 1–3; *srjoint*, scientific rotoscoping joint (=artificial joint). Scale: The chelicera is scaled to 60 cm.

**FIGURE 2:**
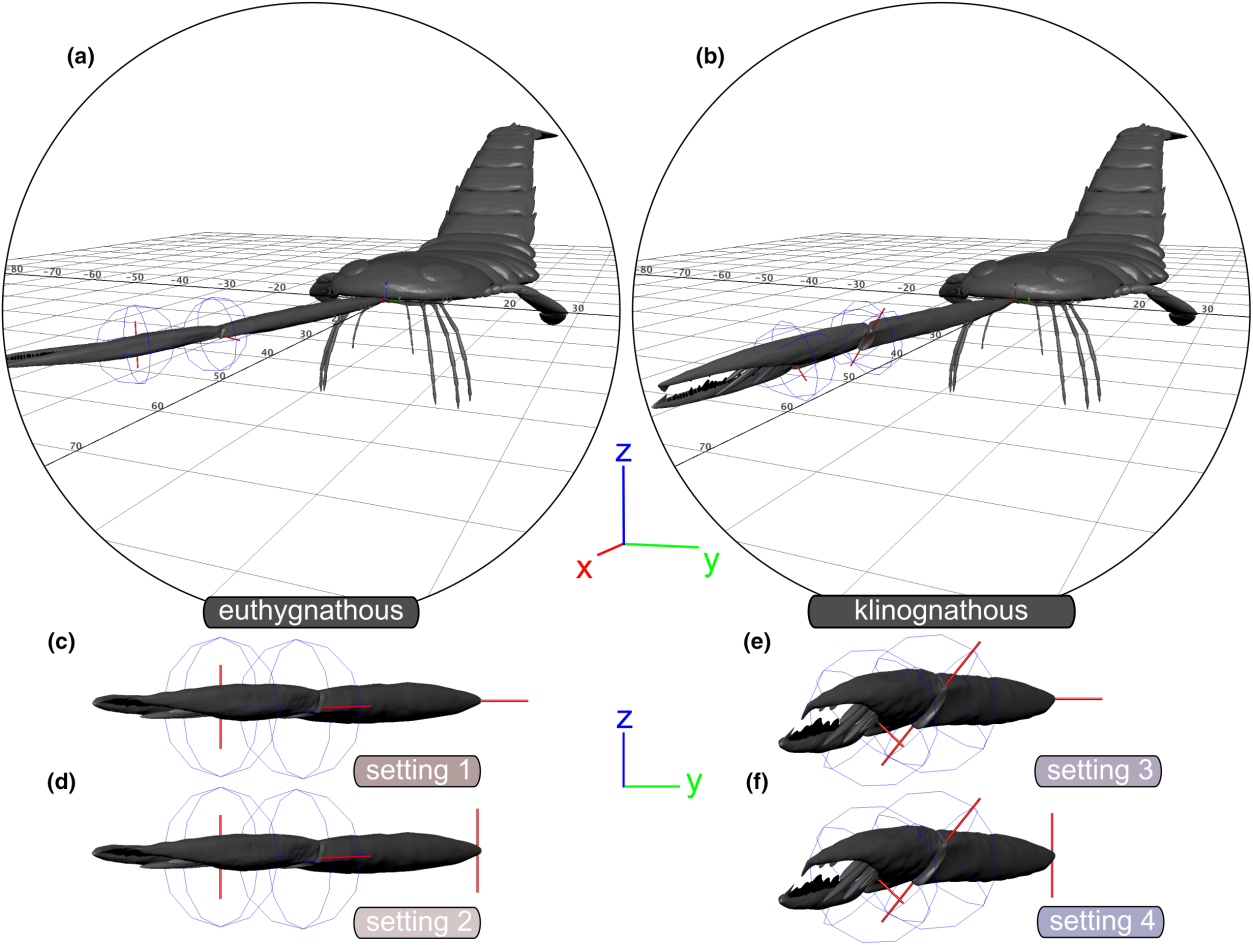
Kinematic settings of the Range of Motion analyses. (a) Kinematic marionette of *Acutiramus* with euthygnathous chelicera (=straight), anterior oblique view. (b) Kinematic marionette of *Acutiramus* with klinognathous chelicera (=rotated 45°), anterior oblique view. (c–f) Kinematic marionettes of the right chelicera representing the four settings, respectively, anterior view. (c) Setting 1: euthygnathous chelicera + horizontal joint axis 1. (d) Setting 2: euthygnathous chelicera + vertical joint axis 1. (e) Setting 3: klinognathous chelicera + horizontal joint axis 1. (f) Setting 4: klinognathous chelicera + horizontal joint axis 1. Scale: Grid units are represented in cm.

Terminology adheres to Clarke and Ruedemann (1912) and Tollerton (1989) (Figure 1a,b). Each chelicera comprises three elements: a basal element, which connects to the epistome/labrum complex on the ventral side of the prosoma (Bicknell, Kenny, & Plotnick, 2023), along with the fixed and free ramus forming the chela (Figure 1c). Each joint is constituted by the proximal and distal cheliceral elements, respectively. These joints are modeled as bicondylar hinge joints, allowing motion in a single plane only (e.g., up and down). Bicondylar joints represent the predominant joint types in arthropod legs (Boxshall, 2004, 2013) and can manifest as either hinge joints or pivot joints (Manton, 1977; Wootton, 1999). The actual orientation angle of the basal cheliceral element into the epistome/labrum complex (referred to as joint 1) remains unknown. We designate this as Chelicera Orientation Angle (COA). To assess the feeding movements and motion capabilities of the chelicera, we employed four kinematic settings of joint 1 articulation (Figure 2; Table 1). For settings with inclined chelicerae (klinognathous), we present them with the free ramus facing more ventrally than dorsally. This aligns with the chelate type of cheliceres observed in Scorpiones and Solifugae, for example, which exhibit orthognathous chelicerae with the free ramus ventrally located. The terminology used is outlined in Table 2.

**TABLE 1:** Kinematic settings.

Setting	Chelicera type	Chelicera Orientation Angle (COA) [°]	Joint axis 1 insertion mode	Enabled motion of the chelicerae
1	Euthygnathous	0	Horizontal	Dorsoventral
2	Euthygnathous	0	Vertical	Mediolateral
3	Klinognathous	45	Horizontal	Dorsoventral
4	Klinognathous	45	Vertical	Mediolateral

*Note*: Euthygnathous chelicerae are defined as being parallel to the longitudinal body axis of the sea scorpion, whereas klinognathous chelicerae are rotated 45° to this axis. For joint axis 1, horizontal insertion into the epistome/labrum complex facilitates dorsoventral movement. Conversely, the vertical insertion of joint axis 1 enables mediolateral movement.

**TABLE 2:** Terminology.

Terminology	Explanation
Arthrodial cavity	Area between elements of an appendage Originally contained soft and flexible arthrodial membrane
COA	Chelicera Orientation Angle [°] 0° in euthygnathous chelicerae 45° in klinognathous chelicerae
el1	Basal cheliceral element, connects the chelicera to the body
el2	Fixed ramus of the chela
el3	Free ramus of the chela
Epistome/labrum‐complex	Articulation side of the chelicerae
Euthygnathous chelicerae	Straight orientation of the chelicerae Chelicerae aligned parallel to the longitudinal body axis Comparable to for instance orthognathous mygalomorph and mesothele Araneae, Amblypygi, Schizomida, Thelyphonida, Opiliones Foelix (2011)
Joint	Flexible construction that links two adjacent elements of an appendage Bicondylar joint: a joint which is made up of two articulation points, limiting elements to movement in just one plane (e.g., up and down) Bicondylar hinge joint: joint type with two articulation points at the same region (either side of the articulation) Bicondylar pivot joint: joint type with two articulation points crossing the lumen Monocondylar joint: joint type with only one articulation point; movement in different planes possible Boxshall (2004, 2013); Manton (1977); Wootton (1999)
Joint axis	Hypothetical construction combining two articulation points of two adjacent elements of an appendage Herein constructed as a cylinder
Klinognathous chelicerae	Diagonal or perpendicular orientation of the chelicerae Chelicerae oriented at an angle to the longitudinal body axis Comparable to for instance labidognathous araneomorph spiders Foelix (2011)
Permutation	Mathematically: permutation with repetition Depending on the number of elements and number of positions Here: 3‐digit sequence of the numbers 1 and 2 (regarding positions 1 and 2), with every sequence occurring only once 2^3^ = 8 (means 2 positions^3 elements^ = 8 permutations)
Position	Translation coordinates inside a 3D space to encode maximum movement in a bicondylar joint (having two positions, e.g., maximum up, maximum down)
ROM	Range of Motion tROM = total Range of Motion (scatter plot) sROM = single Range of Motion (maximum excursion angle in each joint)
Setting	Kinematic set‐ups to explore movement capabilities of the chelicerae
srjoint	Artificial joint which connects the elements Brainerd et al. (2010); Gatesy et al. (2010)

### 2.3 Range of Motion analyses

The kinematic analyses were conducted using Autodesk Maya 2024. Upon importing the .obj file of the three‐dimensional model, a kinematic marionette was constructed following the methodology outlined by Schmidt et al. (2021). This involved assigning hypothetical joint axes and srjoints (Scientific Rotoscoping, Gatesy et al., 2010, as depicted in our Figure 1c) to the corresponding joints, utilizing the X_ROMM tools add‐on (Brainerd et al., 2010). The hypothetical joint axes were geometrically shaped as cylinders to adhere to the most natural mathematical form. Subsequently, a reference ball was affixed to the distal most part of the free ramus. This reference ball tracked the movement of the chelicera, with the x‐, y‐, and z‐translational coordinates of the center of this reference ball serving as a quantification of the Range of Motion.

#### 2.3.1 Positions and permutations

**FIGURE 3:**
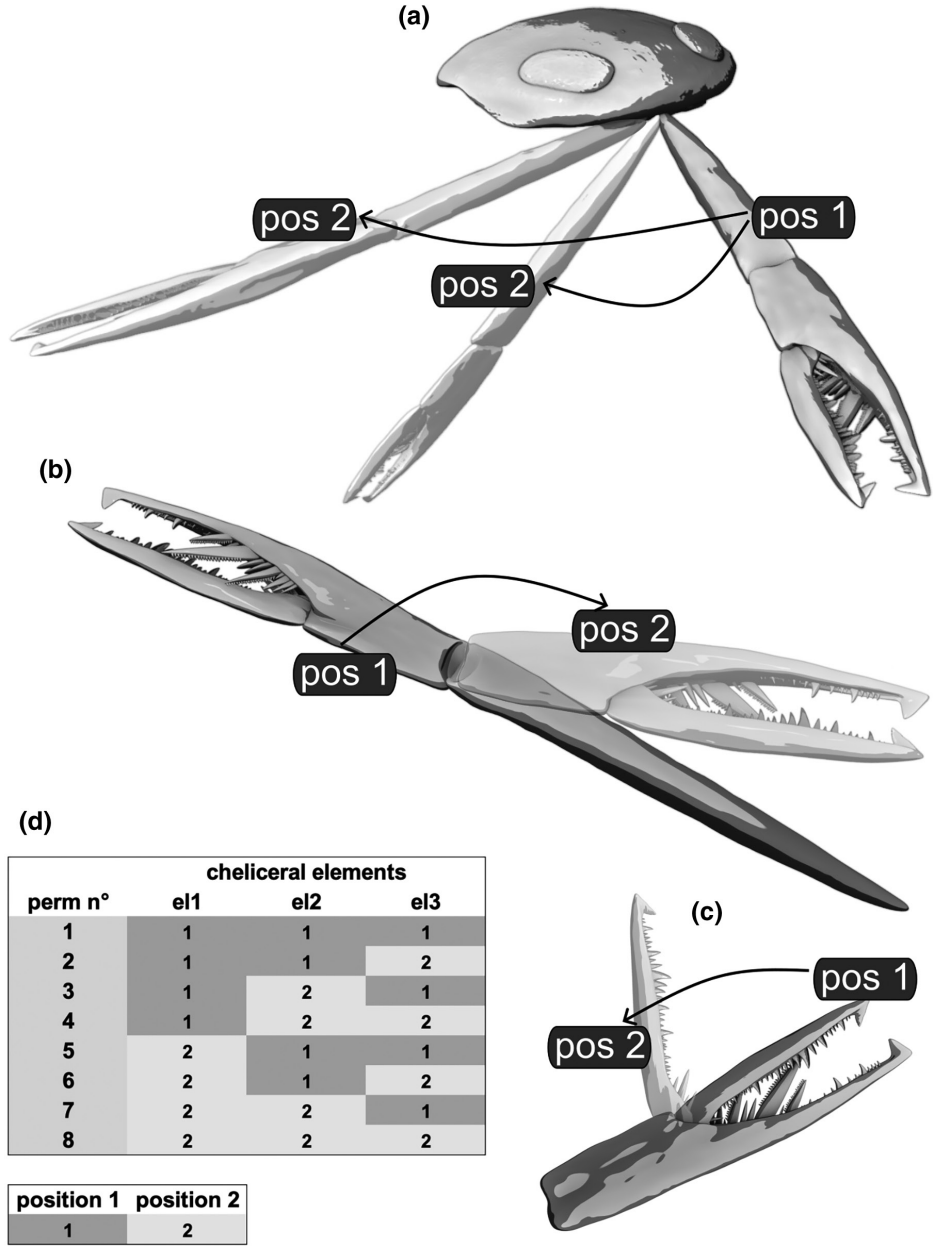
Positions and permutations. (a) Joint 1: Prosoma with right chelicera stretched (pos 1) and rotated (pos 2), either ventrally (Settings 1, 3), or laterally (Settings 2, 4), respectively; anterodorsal oblique view. (b) Joint 2: Fixed ramus stretched (pos 1) or bent (pos 2), ventral oblique view. (c) Joint 3: Chela closed (pos 1) or open with a bent free ramus (pos 2), ventral view. (d) 3‐digit sequence depicting the positions 1 and 2 of the eight permutations utilized for calculating the Range of Motion. Abbreviations: pos = position, perm n° = permutation number, el1 = basal element, el2 = fixed ramus, el3 = free ramus, Scale: The chelicera is scaled to 60 cm.

Being bicondylar, each joint can move only in one plane (df = 1), resulting in only two feasible positions, such as maximum dorsal movement and maximum ventral movement. The entire Range of Motion exists between these two extreme values. The positions were designated as 1 and 2, where position 1 signifies an extended basal element (joint 1), an extended chela/fixed ramus (joint 2), and a closed chela/closed free ramus (joint 3). On the other hand, position 2 entails a flexed basal element (joint 1), a flexed chela/fixed ramus (joint 2), and an opened chela/free ramus (joint 3). With three cheliceral elements (and thus three joints) being movable in two positions (1 and 2), this configuration can be mathematically expressed as eight possible “permutations with repetition” (=2^3^) (Figure 3, also refer to Schmidt et al., 2020, p. 1551, figure 5h). Therefore, there are eight possible maximum motions per chelicera.

#### 2.3.2 Single and total Range of Motion (sROM, tROM)

Analyses of the single Range of Motion (sROM), representing the maximum excursion angle in each joint, followed the methodologies of Schmidt, Melzer, and Bicknell (2022) as well as Schmidt et al. (2020, p. 1551).

Concerning the total Range of Motion (tROM) for the referenced values, the kinematic marionette was configured with the center of the epistome (representing the articulation side of the basal elements of the chelicerae) positioned at the origin of coordinates (forward axis: *X*, up axis *Z*) and scaled to 60 cm (as discussed above). A Range of Motion analysis was conducted following the methodology outlined by Schmidt et al. (2020), involving the completion of all eight permutations per setting. Each permutation, representing a chelicera motion, yielded one data point. In total, for the four settings, this resulted in 16 *x*, *y*, and *z* data points in the scatter plot (reflecting the translational coordinates of the reference ball) per setting (eight permutations for the right chelicera and eight for the left). Data for the right chelicera were computed, whereas data for the left chelicera were obtained by mirroring the y values of the right chelicera. It is important to note that as permutation no. 1–4 of the dorsoventral movement (settings 1, 3) and permutation no. 1–4 of the mediolateral movement (settings 2, 4) are identical, only 24 data points (instead of 32) were collected for the total chelicerae movement with both horizontal (settings 1, 3) and vertical joint axis 1 insertion. The conclusion of ventral and lateral motion was determined when the meshes collided. Dorsal motion of the chelicerae was not modeled, as this would have been impeded by the dorsal shield.

### 2.4 Visualization

Two‐ and three‐dimensional scatter plots as well as the convex hulls were calculated in Python3 (https://www.python.org/; Python Software Foundation. Python Language Reference, version 3.12.2) using PyCharm Community Edition (https://jetbrains.com/pycharm/; JetBrains. PyCharm) with the packages *numpy* (Harris et al., 2020), *matplotlib* (Hunter, 2007) and *scipy* (Virtanen et al., 2020).

## 3 RESULTS

### 3.1 Single Range of Motion (sROM)

The maximum excursion angles in the two distal joints (joints 2 and 3) were not influenced by the chelicera type or the joint axis 1 insertion, hence they are the same in all four settings. For joint 3, being comprised of the fixed ramus and the free ramus, which together form the chela, we measured an sROM of 70° (Table 3). This means that the chela can be opened up to that degree according to our model. Joint 2, the connection between the chela and the elongate basal cheliceral element, allowed a maximum excursion angle of up to 145° till the meshes collided. Contrary to those two joints, the proximal most joint, joint 1, attaching the chelicera to the epistome and thus to the body of the specimen, was influenced by the four settings. With regard to the euthygnathous cheliceral insertion, for setting 1, we measured an sROM of 120°, allowing the chelicera to swing in ventrally. For setting 2, we measured an sROM of 55° for the lateral movement of the chelicera. For the klinognathous chelicera, we measured 109° (setting 3) for an inclined ventral movement of the whole chelicera and 65° (setting 4) for an inclined lateral movement.

**TABLE 3:** Single Range of Motion (sROM).

Chelicera type	Joint	Joint description	sROM [°]	Enabled motion of the joint
Euthygnathous	Joint 3	Fixed ramus‐free ramus‐joint	70	Opening/closing of the chela
	Joint 2	Basal‐cheliceral element‐fixed ramus‐joint	145	Bending of the chela toward basal cheliceral element
	Joint 1	Body‐basal cheliceral element‐joint (horizontal joint axis, setting 1)	120	Dorsoventral movement of the whole chelicera
		Body‐basal cheliceral element‐joint (vertical joint axis, setting 2)	55	Mediolateral movement of the whole chelicera
Klinognathous	Joint 3	Fixed ramus‐free ramus‐joint	70	Opening/closing of the chela
	Joint 2	Basal‐cheliceral element‐fixed ramus‐joint	145	Bending of the chela toward basal cheliceral element
	Joint 1	Body‐basal cheliceral element‐joint (horizontal joint axis, setting 3)	109	Inclined dorsoventral movement of the whole chelicera
		Body‐basal cheliceral element‐joint (vertical joint axis, setting 4)	65	Inclined mediolateral movement of the whole chelicera

*Note*: Maximum excursion angles measured in each joint as well as enabled motion of the respective joint.

### 3.2 Total Range of Motion (tROM)

#### 3.2.1 Setting 1

**FIGURE 4:**
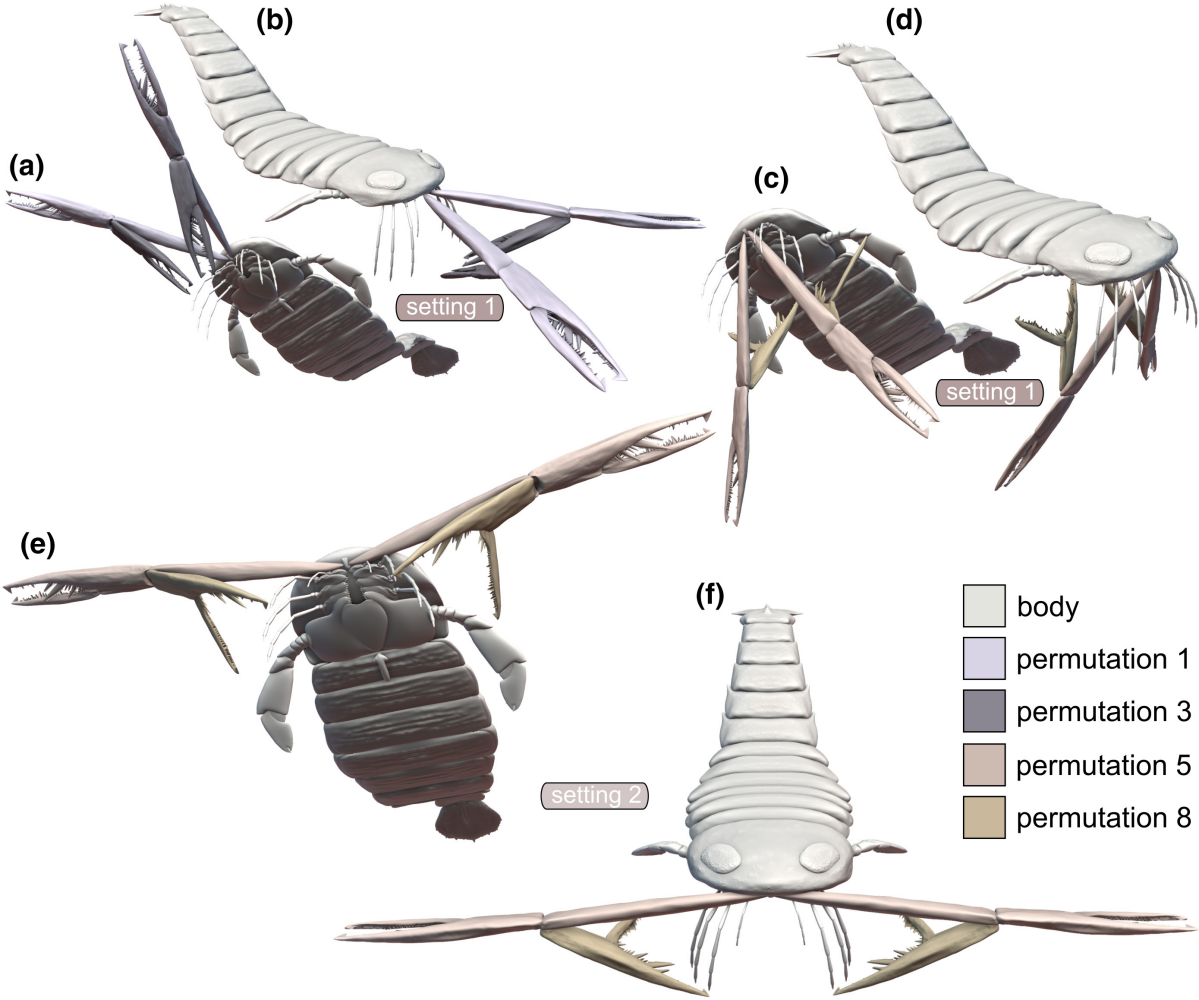
Three‐dimensional reconstruction of *Acutiramus* with euthygnathous chelicerae bent in four different permutations (1, 3, 5, and 8), representing kinematic settings 1 and 2. (a, b) Setting 1, permutations 1 and 3; (a) anteroventral oblique view, (b) anterodorsal oblique view. (c, d) Setting 1, permutations 5 and 8; (c) anteroventral oblique view, (d) anterodorsal oblique view. (e, f) Setting 2, permutations 5 and 8, (e) anteroventral oblique view, (f) anterior oblique view. Note that permutations 1 and 3 for setting 2 are identical to those for setting 1, hence not presented again. Scale: The chelicera is scaled to 60 cm.

**FIGURE 5:**
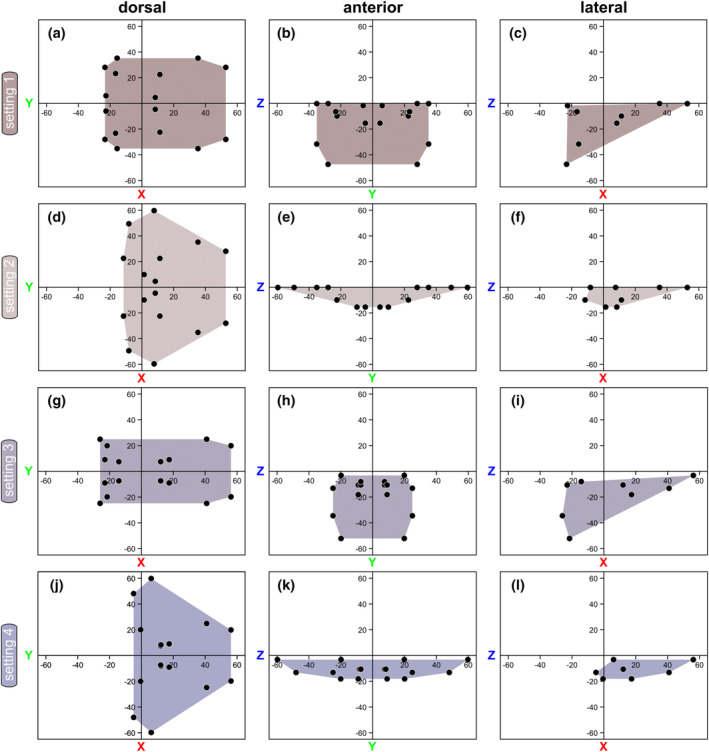
Total Range of Motion (tROM). 2D scatter plots illustrating cheliceral movement for all four settings, encompassing eight permutations per body side, presented in dorsal, anterior, and lateral views, respectively. (a–c) Setting 1; (d–f) Setting 2; (g–i) Setting 3; (j–l) Setting 4. Colored shapes denote the 2D convex hull. Each data point corresponds to the translational coordinates of the center of the affixed reference ball of the free ramus for each permutation. Please note that in (e) and (l), only seven data points per chelicera are visible, as they overlap with another point in the same plane.

*Acutiramus* modeled with euthygnathous chelicerae and a horizontal joint axis 1 would have allowed it to swing in the whole chelicerae ventrally. The tip of the chela and the free ramus would have not gotten very close to the oral region. However, according to joint 1, the chela in its most bent way could have been used to bring food items close enough to the appendages 2–4 for further processing (Figure 4a–d). Nevertheless, even given the maximum excursion in joint 2 would have not enabled the tip of the chela to reach the oral region. For total length measurements, the tip of the chela (the affixed reference ball) maximally reached around 35.14 cm laterally, 47.38 cm ventrally and 52.75 cm anteriorly (Figures 5a–c and 7a,b).

#### 3.2.2 Setting 2

*Acutiramus* modeled with euthygnathous chelicerae and a vertical joint axis 1 would have allowed it to bend the chelicerae laterally. In this kinematic setting, the bent chela and the tip of the free ramus would not get as close to the oral region as in setting 1 (Figure 4e,f).

For total length measurements, the tip of the chela maximally reached around 59.62 cm laterally, 15.4 cm ventrally and 52.75 cm anteriorly (Figures 5d–f and 7a,b).

#### 3.2.3 Setting 3

**FIGURE 6:**
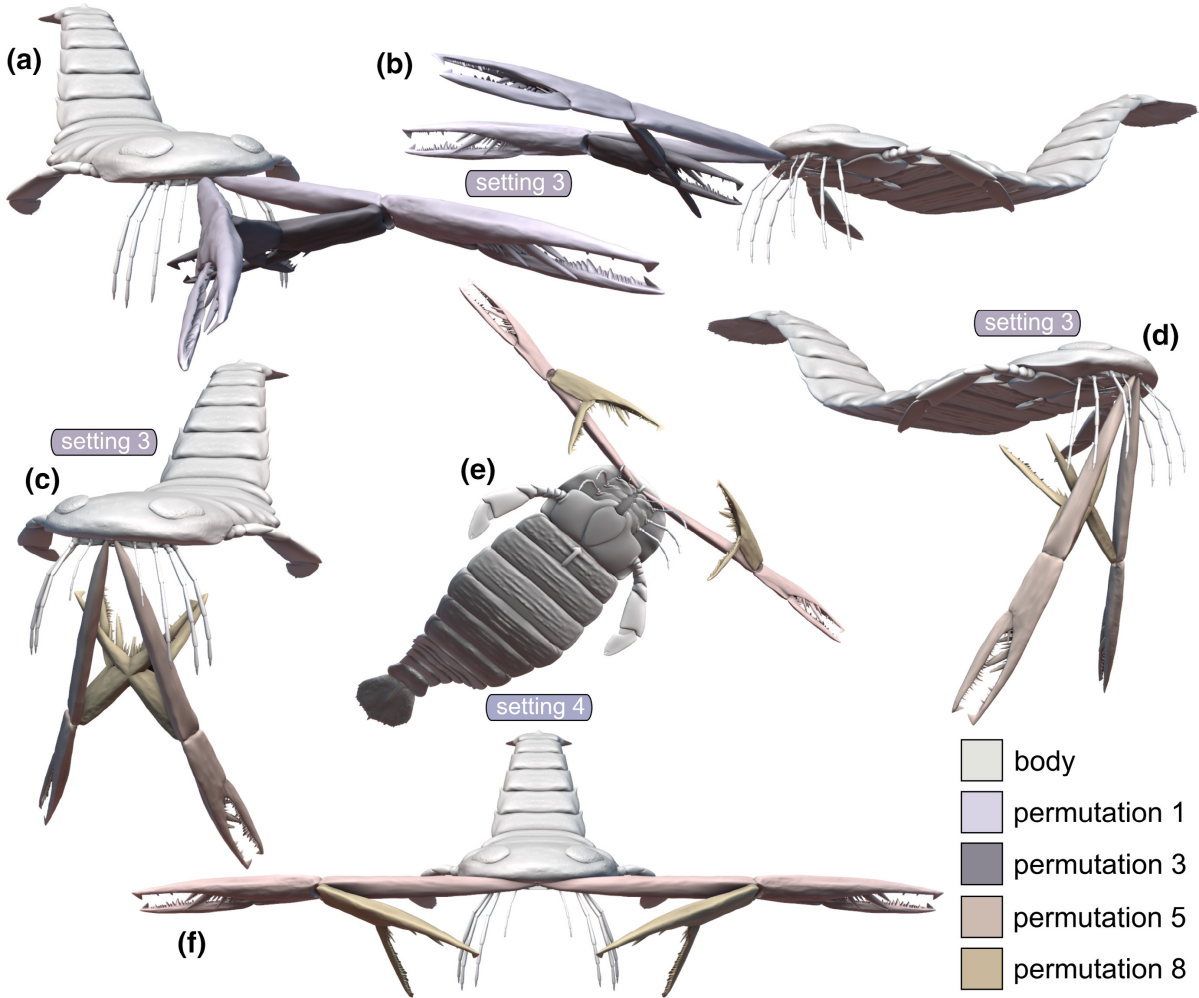
Three‐dimensional reconstruction of *Acutiramus* with klinognathous chelicerae bent in four different permutations (1, 3, 5, and 8), representing kinematic settings 3 and 4. (a, b) Setting 3, permutations 1 and 3; (a) anterodorsal oblique view, (b) anterolateral oblique view. (c, d) Setting 3, permutations 5 and 8; (c) anterodorsal oblique view, (d) anterolateral oblique view. (e, f) Setting 4, permutations 5 and 8, (e) anteroventral oblique view, (f) anterior view. Note that permutations 1 and 3 for setting 4 are identical to those for setting 3, hence not presented again. Scale: The chelicera is scaled to 60 cm.

*Acutiramus* modeled with klinognathous chelicerae and a horizontal joint axis 1 would have allowed it to it to swing in the whole chelicerae ventrally, but inclined. In that manner, both chela could have not been bent totally at the same time, as they would have collided, like in permutation 1 and 3 (Figure 6a,b) or in permutation 5 and 8 (Figure 6c,d). Nevertheless, like in setting 1, the chela in its most bent way could have been used to bring food items close enough to the appendages 2–4 for further processing.

**FIGURE 7:**
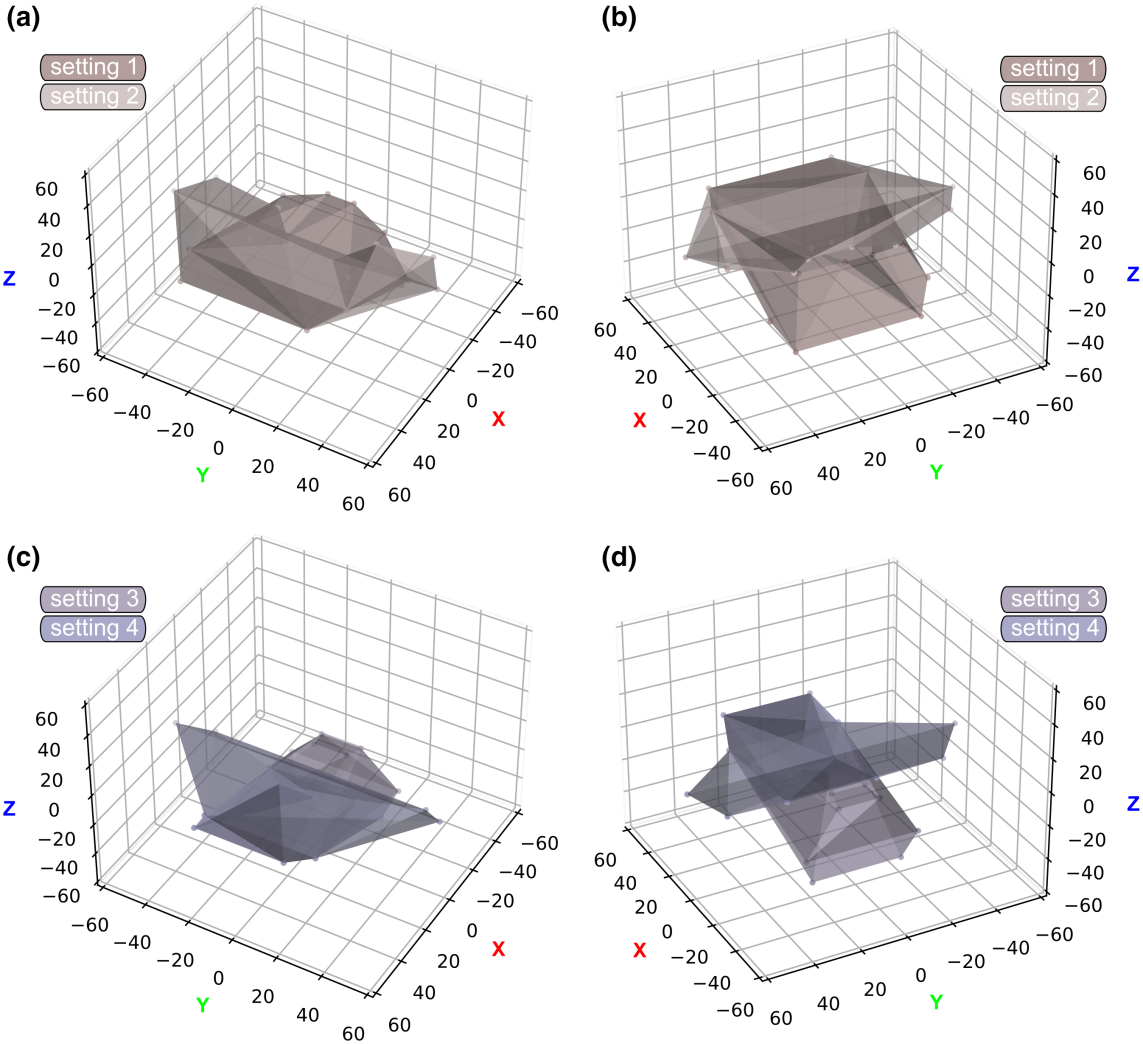
Total Range of Motion (tROM). 3D‐scatter plots illustrating cheliceral movement for all four settings, encompassing eight permutations per body side. Colored shapes denote the 3D convex hulls. Each data point corresponds to the translational coordinates of the center of the affixed reference ball of the free ramus for each permutation. (a) settings 1, 2, anterodorsal oblique view. (b) Settings 1, 2, postero dorsal oblique view. (c) Settings 3, 4, anterodorsal oblique view. (d) Settings 3, 4, postero dorsal oblique view.

For total length measurements, the tip of the chela maximally reached around 24.88 cm laterally, 52.18 cm ventrally, and 56.15 cm anteriorly (Figures 5g–i and 7c,d).

#### 3.2.4 Setting 4

*Acutiramus* modeled with klinognathous chelicerae and a vertical joint axis 1 would have allowed it to bend the chelicerae laterally, but inclined as in setting 3. Also, like in setting 2 before, the bent chela and the tip of the free ramus would not get as close to the oral region as in setting 1 (Figure 6e,f).

For total length measurements, the tip of the chela maximally reached around 59.76 cm laterally, 18.14 cm ventrally and 56.15 cm anteriorly (Figures 5j–l and 7c,d).

All underlying tROM data are presented in the (Table S1).

## 4 DISCUSSION

We investigated four distinct kinematic settings pertaining to the orientation angle of the chelicerae and the insertion mode of joint axis 1, linking each chelicera to the body. Our analysis demonstrated that none of these varied kinematic settings, along with their eight permutations, would have allowed the chelicerae (specifically the tip of the free ramus) of *Acutiramus* to closely approach the oral region. This limitation primarily arises from the length disparities between the basal cheliceral element and the chela, a theoretical proposition previously postulated (Bicknell, Kenny, & Plotnick, 2023; Clarke & Ruedemann, 1912; Kjellesvig‐Waering, 1964; Laub et al., 2010; Størmer, 1955) but never empirically tested. Despite this constraint, in the present study, certain permutations within the four kinematic settings did facilitate the chela to approach the oral region sufficiently—particularly when the chela was fully retracted, as observed in permutation 3 or 8 (Figures 4 and 6). When deliberating on kinematic settings, it becomes crucial to consider the mode that might have been realized in this taxon, emphasizing ecological feasibility, reliability, and comprehensiveness.

### 4.1 Euthygnathous or klinognathous chelicerae?

The disparity in the Range of Motion between euthygnathous (settings 1, 2) and klinognathous (settings 3, 4) chelicerae may vary from subtle to extreme. For instance, if the COA—defined as the angle toward the longitudinal body axis—was very low, only a few degrees, the Range of Motion might not differ significantly. However, as this angle increases, especially reaching a maximum of 90°, the folding of the chela becomes more intricate. We consider the extreme type of klinognathous chelicerae, where two chelicerae face each other with their former ventral sides, highly improbable. Nevertheless, our modeled COA of 45° was a compromise, and it could have been lower or higher (Figure 2a,b). Nonetheless, our modeled klinognathous COA of 45° indicated that the folded chela (permutations 3 and 8, for instance) would have collided with each other (Figure 6a–d).

The true nature of the chelicerae, whether euthygnathous or klinognathous, remains unknown. In nearly all documented pterygotid specimens in the literature, the chelicerae are preserved disarticulated from the body (Bicknell, Kenny, & Plotnick, 2023; Clarke & Ruedemann, 1912; Kjellesvig‐Waering, 1964; Laub et al., 2010; Størmer, 1955). This may be explained by molts, as molting in sea scorpions could have presented several challenges, particularly with the elongated chelicerae in pterygotids, which would pose a particular difficulty (Brandt, 2021; Tetlie et al., 2008).

Concerning the Range of Motion, we would lean toward an euthygnathous chelicera type (settings 1, 2) that might have been realized in *Acutiramus* during that period. This aligns with among the only extant chelicerate group that exhibits elongated chelicerae relative to body size: Opiliones (harvestmen). Within the Dyspnoi suborder, there is a group with montane and troglobiont taxa that employ their elongated chelicerae to prey on small insects and even snails: Ischyropsalidoidea Simon, 1879 (Prieto, 1990; Roewer, 1950; Schönhofer et al., 2015). Despite this dietary approach, elongate chelicerae do also occur as sexual dimorphic characters in males, for instance in the Eupnoi taxa *Pantopsalis* sp. (Taylor, 2004) or in *Neopantopsalis* sp. (Taylor & Hunt, 2009).

### 4.2 Ventral or lateral movement?

The other primary question addressed in this kinematic study was whether the movement of the entire chelicerae was predominantly ventral (settings 1, 3) or lateral (settings 2, 4). The former would be realized if joint axis 1 was horizontally inserted, while the latter would be the case given a vertical insertion of joint axis 1 (Figure 2c–f). Horizontal insertion would have allowed the chelicera to execute a comprehensive ventral movement. For instance, in permutation 5 (Figures 4c,d and 6c,d), this might have been hydro‐dynamically advantageous during swimming. Given the prominence of these large structures, it is plausible that pterygotids somehow folded them ventrally/backward when exhibiting agility (see discussion in Bicknell, Schmidt, et al., 2023). Furthermore, regardless of being euthygnathous or klinognathous, complete flexion of joint 1 and joint 2 would have enabled the chela to approach very close to the oral region for further masticatory work of the gnathobases.

A lateral movement, induced by a vertical insertion of joint axis 1, would result in a wide opening of the chelicerae in both modes (euthygnathous or klinognathous), resembling the action of crabs opening their claws. This scenario would be indicative of a predator hunting for large prey to capture it, similar to what Schmidt, Melzer, and Bicknell (2022) and Schmidt, Melzer, Plotnick, et al. (2022) tested for extant whip spiders, as well as for megalograptid and mixopterid sea scorpions a respectively. However, in both cases, the laterally moved appendages were pedipalps (in the whip spiders) or the appendages III (in the sea scorpions). Therefore, the prey items were grasped by these large structures but further processed by more anterior appendages (such as the smaller, scissor‐ and needle‐like appendages II in the megalograptid and mixopterid sea scorpions) and ultimately by the chelicerae in all taxa.

In Pterygotidae, the enlarged structures are the chelicerae. Consequently, there were no scissor‐ or needle‐like, or even pincer‐like, more anterior appendages for the final food processing and cutting into very small pieces. Therefore, for pterygotids, the primary function of the chelicerae must have been to bring the previously processed and roughly shredded prey items close enough to the ventral side of the prosoma for further processing presumably by the coxae of the other appendages (Bicknell et al., 2018; Poschmann et al., 2017). This was more likely with a ventral movement of the whole chelicera (thus a horizontal joint axis 1 insertion) than a lateral movement. By using state‐of‐the‐art finite element analyses, Bicknell et al. (2022) also demonstrated that the chela of *Acutiramus bohemicus* in contrast to that in the related taxa *Erettopterus bilobus* and *Pterygotus anglicus* was not well‐suited for crushing or processing hard‐shelled prey items. Laub et al. (2010) concluded the same based on mathematical calculations and also suggested that the chelicerae might have been used working in tandem to shred prey (figure 24)–given a lateral movement enabled by joint 1–or to impale prey against the sea floor (figure 23)–given a ventral movement of joint 2.

Ultimately, among all four kinematic settings, based on our calculations, we favor ventral joint 1 movement (thus horizontal joint axis 1) and euthygnathous chelicera (thus parallel to the longitudinal body axis, COA = 0°); hence, setting 1 to us seems the most plausible. As outlined above, extant members of, for example, the harvestmen group Ischyropsalididae Simon, 1879 also have elongate chelicerae, and those can only fulfill a dorsoventral movement (Schönhofer et al., 2015, figure 1a,b,h,i). However, their cheliceral elements are almost of equal lengths, (or the chela even longer than the basal element), allowing their tips to reach their mouth opening, thus not encountering the same problem as pterygotids do.

**FIGURE 8:**
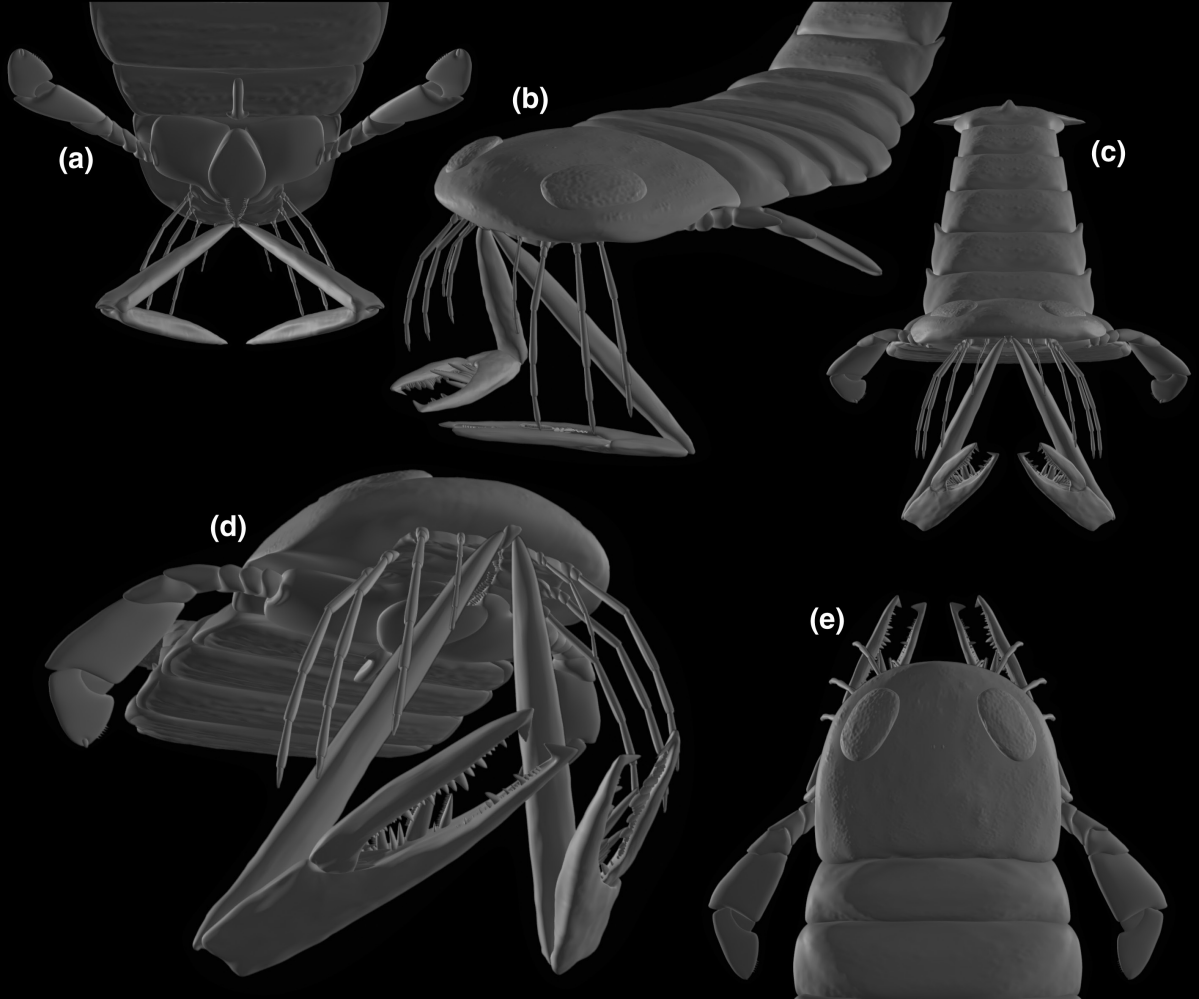
Three‐dimensional reconstruction of *Acutiramus* with modeled chelicerae in a bottom‐up attacking mode. Views from (a) posteroventral, oblique, (b) anterolateral, oblique, (c) frontal, (d) anteroventral, oblique, and (e) dorsal.

Eventually, we intend to explore a concept never brought up yet: the possibility of the chelicerae in *Acutiramus* functioning in a bottom‐up manner rather than the conventional top‐down mechanism (Figure 8). In such a scenario, the basal element would undergo posterior displacement, altering the orientation of joint 2, which connects the chela to the basal element, by 180° compared with our prior analyses of setting 1. Consequently, the chela would now fold dorsally against the basal element instead of ventrally. This intriguing behavior would bear resemblance to extant mantis shrimps (stomatopods), where the second maxillipeds are adapted for smashing or spearing (deVries et al., 2012).

However, in these decapods, the predatory appendages comprise six elements but three, offering a broader range of morpho‐functional and kinematic possibilities. Notably, the striking mechanisms in mantis shrimp smashers and spearers are highly complex (Patek et al., 2013) with the propodus (element 5) being pulled against the merus (element 4) in pre‐strike position and ultimately unleashed for spearing and smashing on high velocity. In *Acutiramus*, we find this prey capture behavior to be improbable, and the idea of this orientation and insertion also never was brought up yet. Although Kjellesvig‐Waering (1964, text figures 1–10) depicted considerable movements of the chelicerae of pterygotids (he posited a four‐segmented chelicera at that time back then), he did not illustrate a bottom‐up attack. *Acutiramus* was generally regarded as a relatively inactive predator due to its limited visual acuity (Anderson et al., 2014; McCoy et al., 2015) and the morphology and dentition of its chelae (Bicknell et al., 2022), suggesting a diet comprising soft or lightly armored prey or scavenged carcasses. Braddy (2023) proposed phyllocarid crustaceans as potential prey based on palaeocommunity structure.

In our Figure 8 model, we utilized the maximum excursion angle (sROM) for both joint 1 (120°) and joint 2 (145°). Following approximately 120° of ventral and posterior motion, the basal element would encounter morphological structures on the ventral side, depending on the type of joint 1. The chela folding against the basal element was also modeled with a measured 145° angle, accounting for factors such as element inflation and structural constraints like tendons and muscles in the living organism. However, even with a higher sROM in joint 2, bringing the chela closer to the basal element, as required in a pre‐strike position (see figure 4 in deVries et al., 2012), prey items would still not be brought close enough to the oral region.

On the contrary, with this mode of articulation and chelicerae orientation, potential prey would be further from the oral region.

In conclusion, we consider the interpretation presented in Figure 8 to be rather improbable, but aimed to illustrate it nevertheless.

### 4.3 Bicondylar or monocondylar joint 1?

After all, the primary question at hand is whether joint 1, the connection between the body and the chelicera, was indeed bicondylar. We modeled it as bicondylar to test the different Range of Motion results. A bicondylar joint (df = 1) is the most common joint type in arthropods, allowing movement in only one plane (Wootton, 1999, figure 1). Conversely, a monocondylar joint would have more degrees of freedom (df > 1), permitting a non‐countable number of permutations. On one hand, from a mathematical and implementation perspective, the conduction and handling of the Range of Motion analyses would be impractical. It would likely result in thousands of permutations. The total number of permutations is calculated based on the number of positions (base) and the number of joints (exponent). Since the base for bicondylar joints is 2 (two positions, either up and down, or left and right, etc.), the number of permutations for bicondylar joints depends solely on the number of joints. Given the tripartite chelicera in *Acutiramus*, we have 2^3^ = 8 permutations. In crab legs, for instance, with six leg elements, we have 2^6^ = 64 permutations (Schmidt et al., 2020, figure 5h), resulting in 64 data points in the scatter plot.

If we were to assume a monocondylar joint, the situation would be different. We would need to define a base greater than 2 (thus df > 1), but small enough to be manageable. For instance, if we assume a monocondylar joint and specify three positions (maximum dorsal, maximum ventral, maximum diagonal, for instance), we would have 3^3^ = 27 permutations for a tripartite chelicera, but ultimately 3^6^ = 729 permutations for a crab leg with six joints. Therefore, for the manual calculation and modeling of Range of Motion with Autodesk Maya, it is more sensible, straightforward, and logical to assume bicondylar joints and consequently calculate with 2^*n*^.

Bicondylar joints are the predominant articulation type in arthropod appendages overall; however, hexapods provide instances of monocondylar joints. In basal hexapods such as Collembola, Diplura, and Protura, mandibles typically are monocondylar. Conversely, more derived insects (Dicondylia) generally possess bicondylar mandibles (von Lieven, 2000). Nevertheless, exceptions exist, demonstrating secondary monocondylie of the mandibles, as observed in hemipterans (Gayubo, 2008) or within the ant superfamily Chalcidoidea (Perfilieva, 2023; van de Kamp et al., 2022).

Furthermore, certain insect leg joints, such as the tibia‐tarsus connection in cockroaches (Blattodea; Pringle, 1938) or the tibia‐basitarsus connection in stoneflies (Plecoptera; Hanson, 1943), are monocondylar. Notably, no extant chelicerate taxon described to date features a monocondylar joint connecting the chelicerae to the body (Foelix, 2011).

It is worth noting that many studies conducted in the past relied on classical preparatory approaches; however, modern investigations, such as micro‐CT or the application of virtual functional kinematics, may potentially reveal earlier results to be inaccurate. For example, in Diplura, Manton (1972) identified several leg joints as monocondylar, but a comprehensive 3D ex vivo re‐study by Hable (2008) analyzing the same species demonstrated them to be either genuinely bicondylar or too intricate to definitively classify.

In Solifugae, the femur‐patella joint is described as monocondylar (Coddington et al., 2004), yet it is acknowledged that this joint has a limited Range of Motion. In this context, “monocondylar” refers to the presence of only one condyle or articulation point, restricting movement to a single plane. This limitation holds true for bicondylar joints with two condyles, as these can be connected with a hypothetical joint axis, enabling movement in only one plane (Wootton, 1999, figure 1). Wootton (1999) accurately summarizes the differences between mono‐ and bicondylar joints, emphasizing that monocondylar joints allow for a higher Range of Motion than bicondylar joints, not a lower.

Additionally, the construction of the joint itself is pivotal in determining the Range of Motion. In *Calanus* sp. (Copepoda), for instance, the body‐coxa joint of each of the four thoracic appendages achieves a maximum excursion angle of approximately 110°, attributed to a specialized sclerotized structure known as the coupler, unique to copepods (Manton, 1977; Perryman, 1961). It is plausible that the body‐coxa joint in pterygotid sea scorpion chelicerae also featured a specialized construction enabling a higher Range of Motion, irrespective of being mono‐ or bicondylar.

Bicknell, Kenny, and Plotnick (2023) proposed, based on the shape of the epistome/labrum complex and the basal cheliceral element, a joint 1 that is somewhat “ball‐and‐socket”‐like. Although they did not explicitly categorize it as monocondylar, they assumed a very high Range of Motion. Our analyses demonstrated that even if a monocondylar joint with an increased Range of Motion was somehow realized, it would have resulted in the same maximum excursion in lateral and ventral orientations, with values in between (Figure 7). Nevertheless, even considering a joint of this type, allowing for a higher Range of Motion, would not have enabled the chela to fully reach the oral region (due to the length differences between the chela and the basal cheliceral element).

Hence, we consider joint 1 to have most likely been a regular bicondylar joint as established in all chelicerates, allowing movement in only one plane but exhibiting a very high Range of Motion and maximum excursion to adequately fold in the entire chelicerae ventrally.

### 4.4 Sexual dimorphism or ontogenetic changes?

The inquiry into whether the elongated chelicerae in Pterygotidae also served as sexual dimorphic characters has not previously been addressed in the literature to our knowledge. Sexual dimorphism among eurypterids has long been recognized and extensively discussed in early studies (Holm, 1898; Schmidt, 1883; Woodward, 1866–1878). However, clear sexual dimorphism primarily pertains to the so‐called genital appendages type A and B (Braddy & Dunlop, 1997; Størmer & Kjellesvig‐Waering, 1969), and which type can be assigned to which sex is also questionable (Selden, 1984). Intrasexual competition, where males compete for access to females, predominates as the primary form of sexual selection in nature (Shuker, 2014). Consequently, males typically exhibit more imposing and conspicuous morphological structures than females (Shine, 1984; Slatkin, 1984). Despite functional kinematic constraints on feeding, the markedly elongated chelicerae may have functioned as display structures, with males possessing longer chelicerae being sexually and genetically more attractive to females, or may have been employed in male–male contests, where the victor gains mating access to the female.

Regarding the condition of the chelicerae, intact structures might be of interest to females according to the handicap theory (Zahavi, 1975), as they would indicate a male's ability to survive and thrive with exaggerated and impractical structures. However, damaged chelicerae could also be intriguing to females as they would signify a male who has engaged in and survived battles. In extant harvestmen, such as *Pantopsalis* sp. or *Fosteropsalis* sp., males exhibit extremely elongated chelicerae (Taylor, 2013), sometimes even proportionally longer in relation to the cephalothorax length compared to Ischyropsalididae.

Nonetheless, speculation is required concerning the actual mating behavior of eurypterids due to the absence of fossil evidence. Additionally, assigning chelicerae definitively to males or females (for the measurement of potential sexual length differences) is challenging, as chelicerae are rarely found fossilized intact with the body, and determining the sex of fossils conclusively is difficult. Furthermore, statistical analysis would require multiple specimens of both sexes with fully elongated chelicerae.

In addition to considering sexual factors, it is imperative to account for ontogenetic differences when examining questions pertaining to conspicuous morphological structures (Gould, 1966, 1977). Ontogenetic analyses have been conducted, for instance, on various eurypterine eurypterids yet (Andrews et al., 1974; Cuggy, 1994; Lamsdell & Selden, 2013).

If juvenile *Acutiramus* sp. (or pterygotids in general) inhabited diverse ecological niches, it is conceivable that the proportion of their chelicerae length to body size may have varied during growth. Indeed, Lamsdell and Selden (2013) demonstrated positive allometric growth for the denticles of the pterygotid sea scorpion *Jaekelopterus howelli* (Kjellesvig‐Waering & Størmer, 1952) over ontogeny. Although they examined 33 specimens, their focus on the denticles allowed for measurements using individual, isolated rami, thus not relying on chelicerae of complete length.

After all, conducting ontogenetic studies on chelicerae growth in pterygotid sea scorpions necessitates not only fossils with fully preserved chelicerae (preferably attached to the prosoma) but also specimens representing various body sizes.

### 4.5 Questions remain

Despite all mentioned above, various unresolved questions could impact cheliceral kinematics and the Range of Motion. One such aspect is the distance between the left and right chelicera or, more precisely, the angle between them. Our 3D model relied on Bicknell, Kenny, and Plotnick, (2023, figure 8C,F). However, if the angle between the chelicerae were lower, bringing them closer when stretched, it might potentially restrict the Range of Motion.

Another factor to consider is the orientation of the joint axis in joint 2: the axis linking the basal cheliceral element to the chela (Figure 1c). According to a conventional interpretation (Clarke & Ruedemann, 1912, pls. 67, 68), in a stretched euthygnathous chelicera, the chela would flex ventrally (Figure 3b), akin to what is observed in *Ischyropsalis* sp. (Schönhofer et al., 2015). However, there is no definitive evidence in the fossil record. One published specimen (YPM IP 018609) illustrates an alternative possibility, where in a stretched euthygnathous chelicera, the chela bends laterally instead of ventrally (Bicknell, Kenny, & Plotnick, 2023, figure 3C). While this configuration might seem plausible, enabling the chelicerae to work cooperatively when processing prey items, the absence of other fossilized chelicerae exhibiting this trait raises the possibility of a taphonomic bias, considering that the chela in that specimen could have disarticulated from the basal cheliceral element after death.

Additionally, the positioning of the free ramus within the chela is a topic of discussion. Some authors (Bicknell, Kenny, & Plotnick, 2023; Braddy, 2023; Braddy et al., 2008; Laub et al., 2010) depicted or modeled them on the exterior side of the chela, while others placed them on the interior side (Clarke & Ruedemann, 1912). However, there is no conclusive fossil evidence, as most chelicerae are preserved apart from the body. Specimen NHMUK PI In 59343 (Kjellesvig‐Waering, 1964, pl. 53, figure 1), featured in Bicknell, Kenny, and Plotnick, (2023, figure 5), portrays another pterygotid (*Erettopterus bilobus*), appearing in ventral view. Although this specimen appears to have an articulated chelicera, since only one is preserved, it is not definitively proven whether it belonged to the left or right side. Assuming it represents the right chelicera, the free ramus would have been located interiorly, and vice versa for the left chelicera. Nonetheless, the authors reconstructed *Acutiramus* with its free rami positioned exteriorly.

We also modeled it exterior, as this closely aligns with the situation in extant *Ischyropsalis* harvestmen (Schönhofer et al., 2015), but also in xiphosurans and scorpions.

Ultimately, the inflation of body parts needs consideration when performing and analyzing the Range of Motion, as different inflation levels could lead to an altered Range of Motion.

## AUTHOR CONTRIBUTIONS

**Michel Schmidt:** Conceptualization (equal); data curation (equal); formal analysis (lead); funding acquisition (supporting); investigation (equal); methodology (lead); project administration (supporting); resources (equal); software (equal); supervision (supporting); validation (equal); visualization (lead); writing – original draft (lead); writing – review and editing (equal). **Roland R. Melzer:** Conceptualization (equal); data curation (equal); formal analysis (supporting); funding acquisition (lead); investigation (equal); methodology (equal); project administration (lead); resources (equal); software (equal); supervision (lead); validation (equal); visualization (supporting); writing – original draft (supporting); writing – review and editing (equal).

## CONFLICT OF INTEREST STATEMENT

No potential conflict of interest was reported by the authors.

## Supporting information


Table S1

## Data Availability

Supplementary 3D kinematic models are available on the Dryad repository: doi: 10.5061/dryad.2fqz612x9 or https://datadryad.org/stash/share/L8GqjkA4yjhlLcStlokINoYYPcgEtcEPRiFS25DFXEw.
